# Evaluation of the effects of pycnogenol (French maritime pine bark extract) supplementation on inflammatory biomarkers and nutritional and clinical status in traumatic brain injury patients in an intensive care unit: A randomized clinical trial protocol

**DOI:** 10.1186/s13063-019-4008-x

**Published:** 2020-02-11

**Authors:** Mahsa Malekahmadi, Omid Moradi Moghaddam, Sheikh Mohammed Shariful Islam, Kiarash Tanha, Mohsen Nematy, Naseh Pahlavani, Safieh Firouzi, Mohammad Reza Zali, Abdolreza Norouzy

**Affiliations:** 1 Student Research Committee, Mashhad University of Medical Sciences, Mashhad, Iran; 2 Nutrition Department, Faculty of Medicine, Mashhad University of Medical Sciences, Bahonar St, Mashhad, Iran; 3 Trauma and Injury Research Center, Critical Care Department, Rasoul-e-Akram Complex Hospital, Iran University of Medical Sciences, Tehran, Iran; 4 Institute for Physical Activity and Nutrition (IPAN), School of Exercise and Nutrition Sciences, Deakin University, Melbourne, Australia; 5 Department of Biostatistics, School of Public Health, Iran University of Medical Sciences, Tehran, Iran; 6 Metabolic Syndrome Research Center, Mashhad University of Medical Sciences, Mashhad, Iran; 7 Behbood Research Center for Gastroenterology and Liver Diseases, Tehran, Iran

**Keywords:** Traumatic brain injury, Critical care, Pycnogenol, Inflammation, Nutrition support, French maritime pine bark extract

## Abstract

**Background:**

Traumatic brain injury (TBI) is one of the major health and socioeconomic problems in the world. Immune-enhancing enteral formula has been proven to significantly reduce infection rate in TBI patients. One of the ingredients that can be used in immunonutrition formulas to reduce inflammation and oxidative stress is pycnogenol.

**Objective:**

The objective of this work is to survey the effect of pycnogenol on the clinical, nutritional, and inflammatory status of TBI patients.

**Methods:**

This is a double-blind, randomized controlled trial. Block randomization will be used. An intervention group will receive pycnogenol supplementation of 150 mg for 10 days and a control group will receive a placebo for the same duration. Inflammatory status (IL-6, IL- 1β, C-reactive protein) and oxidative stress status (malondialdehyde, total antioxidant capacity), at the baseline, at the 5th day, and at the end of the study (10th day) will be measured. Clinical and nutritional status will be assessed three times during the intervention. The Sequential Organ Failure Assessment (SOFA) questionnaire for assessment of organ failure will be filled out every other day. The mortality rate will be calculated within 28 days of the start of the intervention. Weight, body mass index, and body composition will be measured. All analyses will be conducted by an initially assigned study arm in an intention-to-treat analysis.

**Discussion:**

We expect that supplementation of 150 mg pycnogenol for 10 days will improve clinical and nutritional status and reduce the inflammation and oxidative stress of the TBI patients.

**Trial registration:**

This trial is registered at clinicaltrials.gov (ref: NCT03777683) at 12/13/2018.

## Background

### Traumatic brain injury

Traumatic brain injury (TBI) is one of the major health and socioeconomic problems in the world [1]. It is prevalent in both developed and developing societies and affects people of all ages. TBI is called the ‘silent epidemic’ because problems resulting from TBI do not occur immediately [2]. TBI causes about 1.5 million deaths and hospitalizations per year in the USA [3]. TBI is more common among teens and young adult aged 15–45 years, mainly due to road accidents and sport-related events. Men are three times more likely to be injured and more severely damaged [4].

TBI is used instead of ‘head injury’ because it shows the importance of the ‘brain’ [5]. TBI is defined as: ‘An alteration in brain function or physiology due to external force or shock from the outside [6]. Patients with TBI are categorized into three groups based on the Glasgow Coma Scale: mild, moderate, and severe. The Glasgow Coma Scale (GCS) is a system used to assess coma and impaired consciousness [7]. A GCS score of 13–15 is defined as mild, 9–12 as moderate, and 3–8 as severe [7].

The mechanisms of damage to the brain tissue associated with TBI are classified into two categories: primary and secondary. Primary damage is due to the mechanical force involved in the skull and the brain, which seems to be irreversible [8]. The primary injury complications include: brain contusions, axonal injuries, rupturing of blood vessels, and intracranial hemorrhages. Secondary injury complications progress over time [9]. The secondary injury complications include: elevated intracranial pressure, blood–brain barrier (BBB disruption, neuroinflammation, brain edema, cerebral hypoxia, ischemia, and delayed neurodegeneration [10–12].

### Neuroinflammation in TBI

Cytokines, chemokines, and growth factors have proven to play important roles in the pathophysiology of TBI. Immediately after brain injury, proinflammatory cytokines, such as IL-1β, IL-6, and tumor necrosis factor-α (TNF-α) as well as transforming growth factor-beta (TGF-β) are produced in large volume. These worsen the condition of trauma and delay the recovery by producing oxidative stress and matrix metalloproteinases [13, 14]. These post-traumatic inflammatory cascades cause blood–brain barrier (BBB) dysfunction, which ultimately leads to the influx of inflammatory cells from the blood to the brain [15]. Production of reactive oxygen substrates (ROS) directly or indirectly, as oxidative by-products of lipids, proteins, or nucleic acids are common following traumatic brain injury. Malondialdehyde (MDA) is the major by-products of lipid peroxidation. MDA is potentially an atherogenic lipid peroxide and generated in vivo via peroxidation of polyunsaturated fatty acids [16].

### Nutrition support in TBI

Nutritional support is an important issue in intensive care for critically ill patients such as those with TBI. Patients with TBI often remain in a hypermetabolic state where the energy expenditure is increased [17]. Early nutrition support in TBI patients results in a significant reduction in mortality rate, less infectious complications, and lower risk of poor outcome [18]. There has been a growing use of immunonutrition to modulate the inflammatory response in injury or infection and to improve clinical outcomes [19]. Immune-modulation enteral formula has been proven to significantly reduce infection rate in TBI patients [20]. One of the ingredients that can be used in an immunonutrition formula to reduce inflammation and oxidative stress is pycnogenol.

### Pycnogenol

Pycnogenol® (PYC) is recognized as one of the most powerful natural antioxidants, which is a bark extract of the French maritime pine (*Pinus pinaster*) and is rich in flavonoids. The main components of PYC are: polyphenols, specifically mono- and oligomeric units of caffeic acid, ferulic acid, catechin, epicatechin, and taxifolin [21]. It is classified as GRAS (generally recognized as safe) in the USA [22]. Clinical effects of PYC include endothelium-dependent vasodilator activity [23] and anti-thrombotic effect as shown by numerous in vitro and in vivo investigations in animals and human clinical research studies [21, 24]. PYC prevents neurotoxicity and apoptotic cell death in oxidative stress status [25, 26]. Also, PYC protects against lipid peroxidation and pro-oxidants and peroxynitrites [27, 28]. A number of animal studies have proven the protective effect of PYC following traumatic brain injury by suppressing IL-6 and TNF-α levels [29, 30]. No serious adverse effects have been seen in any clinical trials or commercial use [31]. The most commonly observed adverse effect is gastric discomfort due to its mild and transient nature [22].

Recently, we conducted a systematic review and meta-analysis of clinical trials that used PYC in chronic diseases [32]. Our meta-analysis revealed that PYC supplementation may have beneficial effects on glycolipid metabolism by reducing fasting glucose, HbA1c, LDL, and enhancing HDL. Also, PYC reduced CRP, plasma-free radicals, systolic and diastolic blood pressure, and body mass index. In this study, for the first time in the world we aimed to assay the PYC effect on inflammatory markers and clinical status in acute phase in humans.

## Objectives

### Main objective

Previous human studies have reported the neuroprotective and anti-inflammatory effects of PYC. The effect of PYC to reduce neuroinflammation in TBI rat has also been proven. So the main objective of the present study is to study the effect of PYC on the clinical, nutritional, and inflammatory status of TBI patients as the first human study in the world.

### Specific objectives

In achieving this overall goal, numerous issues will be addressed with the specific objective of providing definitive answers to the following questions: (1) Is PYC effective in reducing the inflammatory markers including IL-6, IL-1β, and CRP (C-reactive protein) in TBI patients in an intensive care unit? (2) Is PYC effective in reducing oxidative stress in TBI patients? (3) Is PYC effective in improving the clinical status of TBI patients by improving the APCHE II (Acute Physiology And Chronic Health Evaluation II) (Additional file 1: Table S1) and SOFA (Sequential Organ Failure Assessment) (Additional file 1: Table S2) Score? (4) Is PYC effective in improving anthropometric indices (weight and body composition) and nutritional score measured via NUTRIC score questionnaire? (5) Is PYC effective in reducing 28-day mortality in TBI patients?

### Trial deign

This is a parallel-group randomized trial. Blocked randomization will be used to allocate eligible participants to either the control group or the intervention group. The study framework is superiority.

## Methods/design

### Patient selection

#### Inclusion criteria

All TBI patients admitted directly or transferred to the intensive care units of the participating hospital will be evaluated for eligibility for entry into the randomized clinical trial. Preliminary eligibility criteria are summarized in Table 1.

**Table 1:** Primary criteria for study eligibility

Admission in ICU ^a^ due to TBI ^b^	
18 year ≤ age ≤ 65 year	
GCS ^c^ score > 8	
Stable hemodynamic and metabolic status in the first 24 to 48 h	
Having enteral nutritional support	
Fill out the informed consent form by the patient or first-degree relatives of the patient	

^a^ Intensive care unit. ^b^ Traumatic brain injury. ^c^ Glasgow Coma Scale

#### Exclusion criteria

Patients meeting all preliminary eligibility criteria are considered potentially eligible for the study. Patients are screened for the presence of any specific exclusion criteria which would preclude study entry. These exclusions are designed to eliminate patients for whom participation may be dangerous or patients with serious medical disorders whose impact on operative outcome may obscure the importance of nutritional, clinical, and inflammatory factors. These are summarized in Table 2.

**Table 2:** Exclusion criteria

Pregnancy and lactation	
Morbid obesity: BMI ^a^ ≥ 40	
Failure to start enteral nutrition in the first 24–48 h	
Suffering from autoimmune disorders and HIV/AIDS	
Suffering or having history of cancer and any liver failure	
Receiving positive inotropic medications including dopamine, dobutamine, and epinephrine	
Severe and active bleeding	
Suffering from sepsis	
Having a history of known food allergies	

^a^ Body mass index

#### Default criteria

Once randomization has taken place, patients will be removed from the study only for the following reasons: (1) patient’s or physician’s request, (2) significant change in patient’s treatment process, (3) create any exclusion criteria, (4) sensitivity to PYC supplementation.

#### Sample size

Sample-size calculations were based upon Luzzi et al.ʼs study [33], which showed that the mean CRP change in the treatment group was 60% and the control was unchanged or up to a maximum of 15%. Based on the formula for comparing two proportions of a qualitative attribute from two independent statistical societies, the sample size was determined as 25 individuals in each group, (α = 0.05, β = 0.1, the power of study is 90%). Assuming a probable drop out of the sample, 30 patients in each group will be considered.

A study evaluating the effect of PYC on IL-6 and TNF-α in TBI was an animal study [29] and inappropriate for the human sample-size calculation. Therefore, we used CRP as an inflammatory factor to calculate the sample size. However, for more certainty, we calculated the sample size according to Hakumat Rai et al.ʼs study [17] based on IL-6 change in TBI patients. Based on the mean difference between the two groups for IL-6 and error level of 5% and power of 85%, the total sample size calculated was 46. With a 10% probability of drop out during the study, the total sample size was 50 (25 in each group).

#### Study procedures

The university’s executive committee will oversee the project’s implementation and progress, information security, safety of trial participants, and scientific impact assessment. Also this committee will review data from the trial. The trial sponsor will undertake auditing of the trial procedure.

#### Randomization and masking

We will randomly allocate eligible patients on enrolment [1] to either the control group or the intervention group. The randomization list of unique patient identifiers is generated by the computer-generated random block size site. The classification is based on age (18 to 40 and 40 to 65 years old), gender (male / female) and APACHE II score (0 to 35 and 35 to 71) using quadruple blocks. Nutritionists or clinicians will keep the sealed opaque envelope containing the unique patient identifier and the study group allocation in a locked cabinet in the study laboratory. They will be opened by the second nutritionist. Investigators, all study staff hospital attending clinical teams, and patients will be masked to the study group allocation.

#### Intervention

A pragmatic [34], parallel-group, double-blind, randomized controlled trial (Table 3) will be conducted. We will enroll 60 patients who are admitted to the ICU at a university hospital in Tehran, Iran. All participants or their first-degree relatives will need to provide informed consent to the clinician before participating. Participants will be randomly divided into two groups. The method of randomization and masking are explained above. At the first visit, baseline data will be gathered and the intervention group will receive PYC supplement (OLIGOPIN) in the form oral capsules containing 50 mg French maritime pine bark extract plus 130 mg microcrystalline cellulose. OLIGOPIN powder of each capsule will be dissolved in 10 ml of deionized water and given to patients via gavage (three capsules per day) for 10 days.

**Table 3:** Nine PRECIS-2 domains for trial designing characteristics

Domain	Considerations
Eligibility	All eligible TBI ^a^ patients admitted directly or transferred to the ICU ^b^ of participating hospital will take PYC ^c^ along with their routine treatment
Recruitment path	Pragmatic recruitment through usual visit of patients in ICU
Setting	All tests and evaluation status of patients in this trials are performed routinely as a part of usual care, except specialized test including: IL-6 ^d^, IL-1β ^e^, MDA ^f^, and TAC ^g^
Organization	ICU that has intensivist and its trained stuff
Flexibility in delivery	PYC will be prescribed with gavage
Flexibility in adherence	After the written consent of the patient or his/her companion, PYC will be prescribed by the intensivist and will be gavaged by the nurse. PYC is a dietary supplement and it does not interfere with other medications and will be given as part of the patient’s nutritional formula
Follow-up	Usual follow-up that is performed for critically ill patients will be done in this trial; also, a 28-day follow-up for mortality will be performed
Primary outcome	All outcome directly are related to clinical status of patients
Primary analysis	Intention-to-treat with all available data will be used for analysis

^a^ Traumatic brain injury. ^b^ Intensive care unit. ^c^ Pycnogenol. ^d^ Interleukin-6. ^e^ Interleukin-1β. ^f^ Malondialdehyde. ^g^ Total antioxidant capacity

The control group will receive oral capsules containing 130 mg microcrystalline cellulose with 10 ml of deionized water via gavage (three capsules per day) for 10 days.

The capsules will be given by the investigator to the patients by gavage, so fidelity to the intervention will be strong. However, for more certainty, at the end of each day, the number of capsules remaining for each patient will be checked. In order to control the confounding effect of food intake, both the control group and the intervention group receive the standard formulas based on their daily required energy via enteral root feeding.

#### Possible risk assessment of intervention

Initially, an intervention with a dose of 150 mg of PYC will be started for ten patients, and in the absence of clinical complications and observing the expected effect on the reduction of inflammatory markers, the same dose will be continued. Otherwise, it will be reduced to 100 mg, if there is any adverse effect. There have been no reports of serious adverse events in any clinical trials or commercial use of OLIGOPIN. However, these patients will be regularly evaluated biochemically and clinically each day, and the liver function tests including serum levels of ALT (alanine aminotransferase) and AST (aspartate aminotransferase) will be checked. If there are any potential complications from intervention or if the physician determines that the intervention should be discontinued, the supplements will be immediately removed from the patient’s enteral nutrition.

#### Data collection

Data will be collected at four main times: at baseline, at the 5th day of intervention, at the 10th day of intervention, and at the 28-day follow-up visit. At baseline, demographic characteristics are gathered via a questionnaire. Anthropometric assessment including height (via ulna length), weight (by using portable scale “Balas”), body mass index and body composition (by using bio impedance device “Inbody”) will be measured at baseline, the 5th day, and at the end of the intervention.

**Fig. 1:**
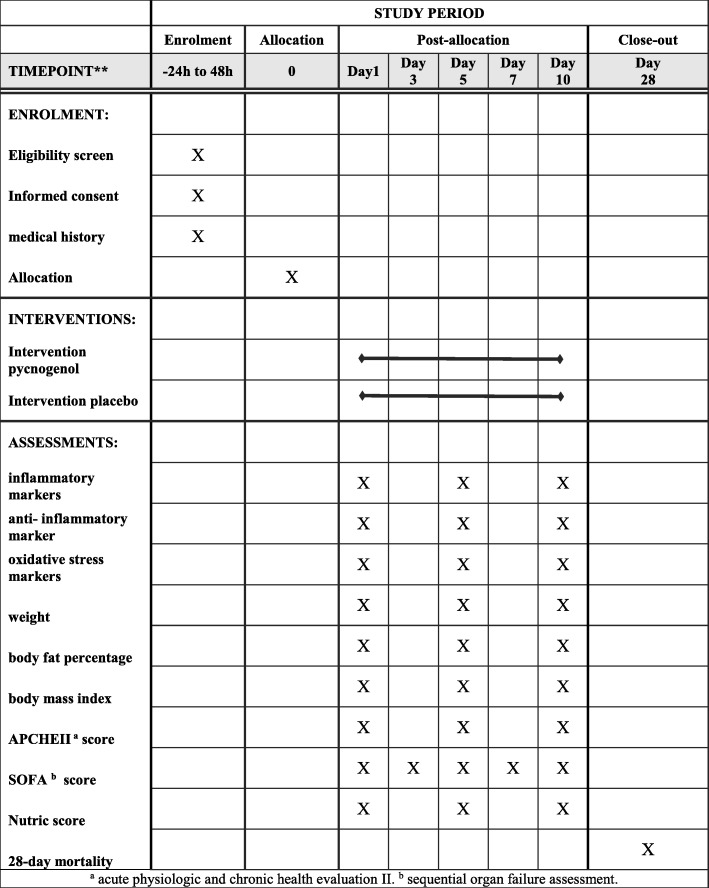
SPIRIT diagram of recommended content for the schedule of enrolment, interventions, and assessments

In order to evaluate inflammatory and oxidative stress markers, 10 cc of venous blood will be taken from each patient at the baseline, at the 5th day, and at the end of the study. The serum sample will be isolated and used to measure the markers via ELISA kits. APACHE ІІ (for assessment of clinical status of patients) and NUTRIC questionnaires (for assessment of nutritional status) will be filled out at the base line, 5th day, and the end of study. The SOFA questionnaire (for assessment of organ failure) will be filled out every other day. The mortality rate will be asked by phone within 28 days of the start of the intervention. A SPIRIT diagram of the recommended content for the schedule of enrolment, interventions, and assessments is shown in Fig. 1.

#### Data management

Specially designed forms will be completed by study staff at each time point, and scanned, verified, and committed to a local site database within 48 h of completion. Completed forms will be stored as the source documentation in a locked cabinet, with access restricted to specified study team members. The forms will be identified by a unique participant ID number and will not contain any patient identifiable information. Queries based on data in the database will be generated daily, including date, range, and logic checks.

### Outcomes

The measurable outcomes are summarized in Table 4.

**Table 4:** Measurable outcomes

Outcome	Time frame	Measurement method
Change of inflammatory markers: IL-6 ^a^, IL-1β ^b^	5 and 10 days	ELISA kit
Change of inflammatory marker: CRP ^c^	5 and 10 days	Auto analyzer
Change of oxidative stress markers: MDA ^d^, TAC ^e^	5 and 10 days	Calorimetry^*^
Change of weight	5 and 10 days	Portable scale “Balas”
Change of body fat percentage	5 and 10 days	Bio impedance device “Inbody”
Change of body mass index	5 and 10 days	Equation
Change of APCHE ІІ ^f^ score	5 and 10 days	APCHEІІ score questionnaire
Change of SOFA ^g^ score	3, 5, 7, 9, 10 day	SOFA score questionnaire
Change of NUTRIC score	5 and 10 days	NUTRIC score questionnaire
28-day mortality	28 days	Telephone follow-up

^a^ Interleukin-6. ^b^ Interleukin-1β. ^c^ C-reactive protein. ^d^ Malondialdehyde. ^e^ Total antioxidant capacity ^f^ Acute physiologic and chronic health evaluation II. ^g^ Sequential organ failure assessment

*Colorimetry is a scientific technique that is used to determine the concentration of colored compounds in solutions by the application of the Beer–Lambert law, which states that the concentration of a solute is proportional to the absorbance

### Statistical methods

The trial profile will be summarized using a CONSORT flow chart, including reasons for non-eligibility and non-enrolment [35]. The objective of this clinical trial is to determine if PYC supplementation improves clinical and nutritional outcomes in TBI patients admitted to an ICU or not. To answer this question, the outcomes of patients receiving PYC supplements will be compared with the outcomes of patients receiving placebo.

**Fig. 2:**
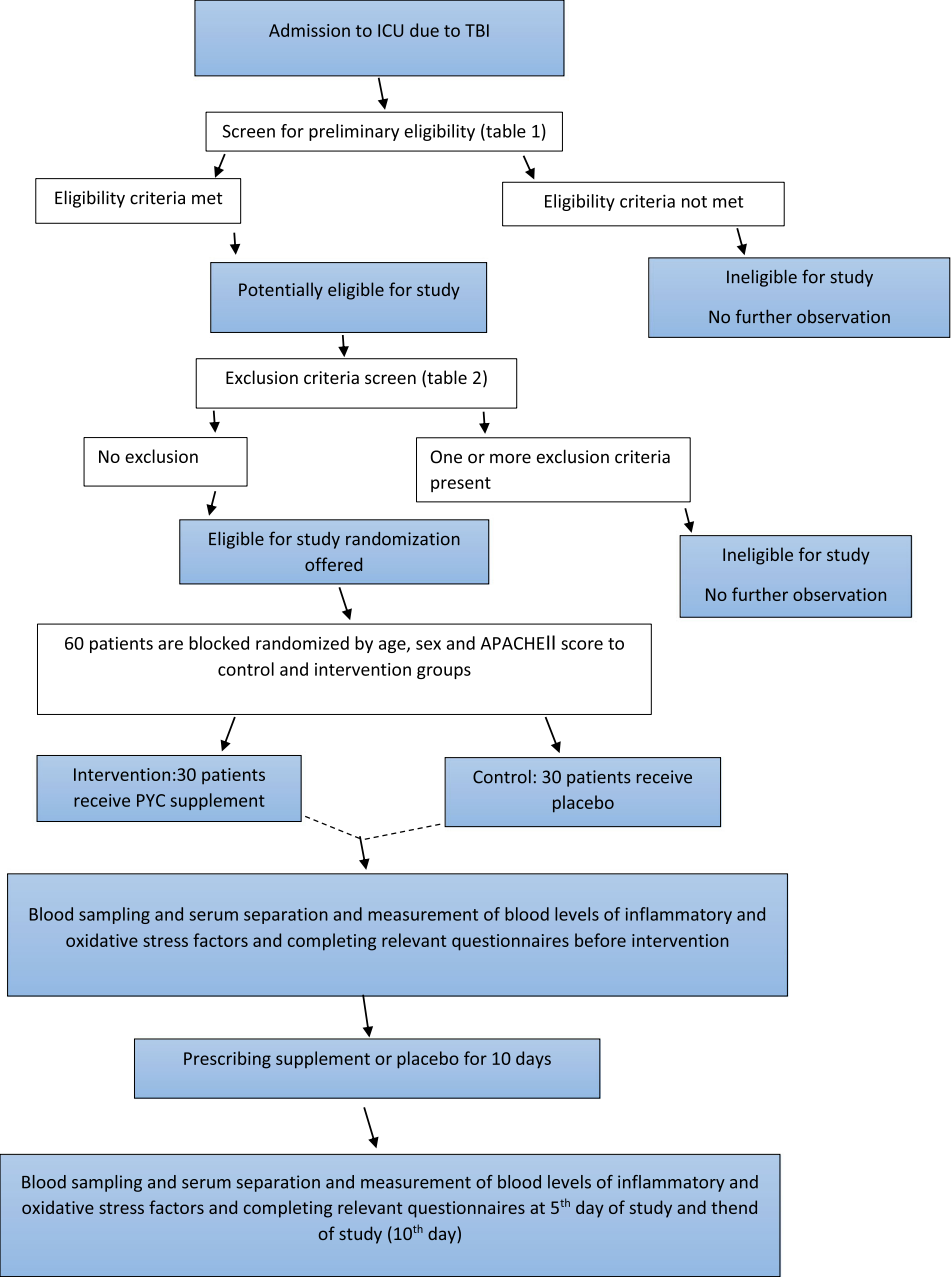
Study design flow diagram

All analyses will be conducted by initially assigned study arm in an intention-to-treat analysis, and adjusted for randomization site. Thus, all randomized patients who will receive at least one dose of study treatment and who will have both a baseline and at least one post-baseline measurement will be analyzed. The data will be expressed as mean ± SD. Statistical analyses will be conducted with SPSS version 19 (SPSS Institute, Chicago, IL, USA). Chi-square test will be done for categorical variables. *t* test will be done to assess the statistical significance of the continuous variables. Comparable nonparametric test (Mann–Whitney *U* test) will be substituted when tests for normality and equal variance failed. A value of *p* = 0.05 will be used as a criterion for statistical significance. Survival analysis will be performed with log-rank test. The study design flow diagram is summarized in Fig. 2. More details about the statistical analyses plan is presented in Additional file 2.

## Discussion

The purpose of this trial is to establish proof of concept of the efficacy of Oligopin in patients with TBI (GCS score > 8). Patients will be screened and randomly enrolled into the intervention and control groups based on age, gender, and APACHE II score. Adding Oligopin to a nutritional formula might reduce neuroinflammation and oxidative stress and improve clinical and nutritional status in TBI patients. However, human study about PYC (Oligopin) in this patient group has not been done so far.

It is assumed that oxidative stress mediated through the superoxide radical (superoxide) and other reactive oxygen species (ROS) may be principal to inflammation and impaired neural function [36]. The acute inflammatory response differs in early and late stages of TBI; too much inflammation for too long delays recovery [37]. Shortly after brain injury, there is mass production of proinflammatory cytokines, such as IL-1β, IL-6, and CRP [38].

In the event of TBI, IL-1β is the most studied cytokine. Glial cells produce IL-1β and affect neurons and other brain cells. IL-1β motivates inflammatory responses and aggregates immune cells, disrupts the BBB, and forms edema, and leads to loss of neurons [39]. The high level of IL-1β has been detected in CSF and brain tissue within the early hours of a brain injury in humans as well as in experimental animals [39]. Administration of anti-IL-1β antibodies decreased edema and degradation of brain tissue. A previous study reported improvement of cognitive function in rats following TBI [40]. There are similar findings for IL-6. Intervention to mitigate IL-6 in animals with mild TBI triggers normal brain function and reduces the effects of hypoxia (aggravation of inflammation of brain damage) [41]. In TBI patients, CRP levels are correlated with the duration of hospitalization in an ICU and dependence on a ventilator, and the severity of the damage [42]. Finally, we selected these inflammatory factors as outcomes of the study.

### Duration of intervention

In this study, we selected 10 days for intervention. According to previous studies, the odds of survival in the first 10 days of admission of the patients in the ICU have a declining slope and after 10 days the slope of the decline will be milder [43, 44]. Therefore, any intervention of treatment in this period (the first 10 days), which leads to a reduction in the risk of mortality, has great importance. On the other hand, the duration of intervention used in clinical trials to evaluate the clinical effects of PYC supplementation has varied from several hours to several months [22, 45, 46]. So in this study we expect to see the expected effects after 10 days.

### Dose of supplementation

The average dose used in most human studies that has beneficial effects in improving inflammation is 150 mg [22, 47, 48]. No side effects have been reported with this dose. Therefore we chose 150 mg Oligopin for this study.

### Trial status

This trial is registered at clinicaltrials.gov (ref: NCT03777683) on December 17, 2018, and is ongoing. It is the first version of the protocol. In April 2019, recruitment began, and the anticipated date to complete the study is February 2020.

## Supplementary information

**Additional file 1: Table S1.** APCHE II (Acute Physiologic and Chronic Health Evaluation II) score content. **Table S2.** SOFA (Sequential Organ Failure Assessment) score content.

**Additional file 2.** Statistical analysis plan.

## Data Availability

Final study datasets will be stored locally and securely at Trauma and Injury Research Center, Iran University of Medical Sciences, Tehran, Iran, for long-term storage and access. Participant-level data will be made available by request on a case-by-case basis. All Principal Investigators will access to the data sets. To ensure confidentiality, data dispersed to project team members will be blinded of any identifying participant information.

## Abbreviations

ALT: Alanine aminotransferase
APACHE II: The Acute Physiology and Chronic Health Evaluation
AST: Aspartate aminotransferase
BBB: Blood–brain barrier
CRP: C-reactive protein
IL-1β: Interleukin-1β
IL-6: Interleukin-6
MDA: Malondialdehyde
NUTRIC score: Nutrition assessment in critically ill
PYC: Pycnogenol
ROS: Reactive oxygen substrates
SOFA: The Sequential Organ Failure Assessment
TAC: Total antioxidant capacity
TBI: Traumatic brain injury
TGF-β: Transforming growth factor-beta
TNF-α: Tumor necrosis factor-α
