# Supplementary material for: Evaluation of the effects of pycnogenol (French maritime pine bark extract) supplementation on inflammatory biomarkers and nutritional and clinical status in traumatic brain injury patients in an intensive care unit: A randomized clinical trial protocol
Source: Trials. 2020 Feb 11;21:162. doi: 10.1186/s13063-019-4008-x (PMC7014642; doi:10.1186/s13063-019-4008-x)
Supplement: Supplementary file 1 — Additional file 1: Table S1. APCHE II (Acute Physiologic and Chronic Health Evaluation II) score content. Table S2. SOFA (Sequential Organ Failure Assessment) score content. [file 13063_2019_4008_MOESM1_ESM.docx]

**Legend of supplementary tables**

**Table S1** APCHE II (Acute Physiologic and Chronic Health Evaluation II) score content

**Table S2** SOFA (Sequential Organ Failure Assessment) score content

| **Table S1. APCHE II (Acute Physiologic and Chronic Health Evaluation II) score** | | | | | | | | | |
| --- | --- | --- | --- | --- | --- | --- | --- | --- | --- |
| Physiologic Varible | +4 | +3 | +2 | +1 | 0 | +1 | +2 | +3 | +4 |
| Temperature | ≥41 | 39-40.9 |  | 38.5-38.9 | 36 -38.4 | 34-35.9 | 32-33.9 | 30-31.9 | <29.9 |
| Mean Arterial Pressure - mm Hg | >160 | 130-159 | 110-129 |  | 70-109 |  | 50-69 |  | <49 |
| HeartRate (ventricular response) | >180 | 140-179 | 110-139 |  | 70-109 |  | 55-69 | 40-54 | <39 |
| Respiratory Rate  (non-ventilated or ventilated) | >50 | 35-49 |  | 25-34 | 12-24 | 10-11 | 6-9 |  | <5 |
| Oxygen delivery (ml/min) or PaO_2_ (mm Hg) | >500 | 350-499 | 200-349 |  | <200   PO2>70 | PO2 61- 70 |  | PO2 55- 60 | PO2<55 |
| Arterial pH (preferred) | >7.7 | 7.6-7.69 |  | 7.5- 7.59 | 7.33- 7.49 |  | 7.25 -7.32 | 7.15 -7.24 | <7.15 |
| Serum HCO3 (venous mEq/l)  (not preferred, but may use if no ABGs) | >52 | 41-51.9 |  | 32-40.9 | 22-31.9 |  | 18-21.9 | 15-17.9 | <15 |
| Serum Sodium(mEq/l) | >180 | 160-179 | 155-159 | 150-154 | 130-149 |  | 120-129 | 111-119 | <110 |
| Serum Potassium(mEq/l) | >7 | 6 -6.9 |  | 5.5-5.9 | 3.5-5.4 | 3 -3.4 | 2.5 -2.9 |  | <2.5 |
| Serum Creatinine (mg/dl)  Double point score for acute renal failure | >3.5 | 2 -3.4 | 1.5 -1.9 |  | 0.6 - 1.4 |  | <0.6 |  |  |
| Hematocrit (%) | >60 |  | 50-59.9 | 46-49.9 | 30- 45.9 |  | 20 -29.9 |  | <20 |
| White Blood Count (total/mm3)  (in 1000s) | >40 |  | 20- 39.9 | 15 -19.9 | 3- 14.9 |  | 1- 2.9 |  | <1 |
| A. Total Acute Physiology Score (sum of 12 above points) | | | | | | | | | |
| B. Age points (years) <44=0; 45 to 54=2; 55 to 64=3; 65 to 74=5; >75=6 | | | | | | | | | |
| C. Chronic Health Points (see below) | | | | | | | | | |
| Total APACHE II Score (add together the points from A+B+C) | | | | | | | | | |

Chronic Health Points: If the patient has a history of severe organ system insufficiency or is immunocompromised as defined below, assign points as follows: 5 points for non-operative or emergency postoperative patients 2 points for elective postoperative patients

**Interpretation of Score:**

| Score | Death Rate (%) |
| --- | --- |
| 0-4 | 4 |
| 5-9 | 8 |
| 10-14 | 15 |
| 15-19 | 25 |
| 20-24 | 40 |
| 25-29 | 55 |
| 30-34 | 75 |
| >34 | 85 |

| **Table S2. SOFA (Sequential Organ Failure Assessment) score content** | | | | | |
| --- | --- | --- | --- | --- | --- |
| Organ System, Measurement | SOFA score | | | | |
|  | 0 | 1 | 2 | 3 | 4 |
| Respiration PaO_2_/FiO_2_, mmHg | Normal | <400 | <300 | <200  (with respiratory support) | <100  (with respiratory support) |
| Coagulation Platelets  x10^3^ /mm^3^ | Normal | <150 | <100 | <50 | <20 |
| Liver Bilirubin,mg/dL (µmol/l) | Normal | 1.2-1.9  (20-32) | 2.0-5.9 (33-101) | 6.0-11.9  (102-204) | >12.0  (<204) |
| Cardiovascular Hypotension | Normal | MAP<70  mmHg | Dopamine≤ 5 or dobutamine (any dose)** | Dopamine >5 or epinephrine ≤0.1 or norepinephrine ≤ 0.1 | Dopamine >15 or epinephrine > 0.1 or norepinephrine > 0.1 |
| Central Nervous System Glasgow Coma Score | Normal | 13-14 | 10-12 | 6-9 | <6 |
| Renal Creatinine, mg/dL (µmol/l) or  Urine output | Normal | 1.2-1.9 (110-170) | 2.0-3.4  (171-299) | 3.5-4.9  (300-440)  or <500 mL/day | >5.0  (>440)  or <200 mL/day |

**Interpretation of Score:**

| Maximum SOFA Score | Mortality |
| --- | --- |
| 0 to 6 | < 10% |
| 7 to 9 | 15 - 20% |
| 10 to 12 | 40 - 50% |
| 13 to 14 | 50 - 60% |
| 15 | > 80% |
| 15 to 24 | > 90% |
