# Supplementary material for: Evaluation of the effects of pycnogenol (French maritime pine bark extract) supplementation on inflammatory biomarkers and nutritional and clinical status in traumatic brain injury patients in an intensive care unit: A randomized clinical trial protocol
Source: Trials. 2020 Feb 11;21:162. doi: 10.1186/s13063-019-4008-x (PMC7014642; doi:10.1186/s13063-019-4008-x)
Supplement: Supplementary file 2 — Additional file 2. Statistical analysis plan. [file 13063_2019_4008_MOESM2_ESM.docx]

**Statistical Analysis Plan**

**(SAP)**

**INTRODUCTION**

This project aims to test in a single-center randomized clinical trial (RCT) if pycnogenol supplementation in subjects with traumatic brain injury (TBI) resident in intensive care unit can reduce inflammation and oxidative stress factor (primary outcome measure), and improve clinical and nutritional status(secondary outcome measures).

This statistical analysis plan (SAP) will give more detailed descriptions of the endpoints in the study and the corresponding analyses.

**DATA SOURCE**

Specially designed forms will be completed by study staff at each time point, and scanned, verified and committed to a local site database within 48 h of completion. Completed forms will be stored as the source documentation in a locked cabinet, with access restricted to specified study team members. The forms will be identified by a unique participant ID number and will not contain any patient identifiable information. Queries based on data in the database will be generated daily, including date, range and logic checks.

**ANALYSIS OBJECTIVES**

To study if pycnogenol supplementation in patients with TBI in intensive care unit can reduce inflammatory markers (C-reactive protein, IL-6, and IL-1β), improve stress oxidative biomarkers (malondialdehyde and total antioxidant capacity), clinical status (APACHE and SOFA score) and nutritional status (Nutric score).

**ANALYSIS SETS/ POPULATIONS/SUBGROUPS**

All TBI patients admitted directly or transferred to the intensive care units of participating hospital are evaluated for eligibility for entry into the randomized clinical trial.

| Table 1. primary criteria for study eligibility |
| --- |
| Admission in ICU ^a^ due to TBI ^b^ |
| 18 year ≤ age ≤ 65 year |
| GCS ^c^ score ˃ 8 |
| Stable hemodynamic and metabolic status in the first 24 to 48 hours |
| Having enteral nutritional support |
| Fill out the informed consent form by the patient or first-degree relatives of the patient |
| ^a^ Intensive care unit. ^b^ Traumatic brain injury. ^c^ Glasgow coma scale. |

| Table 2. exclusion criteria |
| --- |
| Pregnancy and lactation |
| Morbid obesity: BMI ^a^ ≥ 40 |
| Failure to start enteral nutrition in the first 24-48 hours |
| Suffering from autoimmune disorders and HIV/Aids |
| Suffering or having History of cancer and any liver failure |
| Receiving positive inotropic medications including Dopamine, Dobutamine and Epinephrine |
| Severe and active bleeding |
| Suffering from Sepsis |
| Having a history of known food allergies |
| ^a^ Body mass index. |

Intention-to-treat (ITT): All randomized study subjects. This will be seen as the primary population for the analysis.

The primary variables is often systematically related to other influences apart from treatment. For example, there may be relationships to covariates such as age and sex, body mass index, type of medications and surgery. Therefore subgroup analysis will be used.

**ENDPOINTS AND COVARIATES**

The primary analysis will compare intervention groups (pycnogenol supplementation vs control treatment) on their mean change in inflammatory and stress oxidative biomarkers (malondialdehyde and total antioxidant capacity) between baseline and 5 and 10 days using a linear mixed model. The Difference in inflammatory and stress oxidative biomarkers from baseline to time points where it is measured during the study (5 and 10 days) will be the dependent variable. Dietary intake at baseline and medication therapy will be included as covariates. The estimated difference in mean change from baseline to 10 days and the corresponding 95 % confidence interval (CI) will be presented.

APACHE, SOFA, and Nutric score, weight, body mass index, body fat percent and mortality will be analyzed using the same method as for the primary outcome, including usage of the baseline value for the actual factor as a covariate.

**HANDLING OF MISSING VALUES AND OTHER DATA CONVENTIONS**

If missing for primary or secondary outcomes are less than 5% a complete case analysis without imputing missing values will be done. If missing data are more than 5% Little’s test will be done. If the test demonstrates that the complete data set is a random sample we will analyze without imputing missing values. If Little’s test demonstrates that the complete data set is not a random sample of the total data set we will report the point estimates and their 95% confidence limits by using a worst/best scenario imputation. If the worst/best scenario allow for the same conclusion we will not conduct multiple imputations for the missing values. However, if the worst/best scenario imputation indicates different conclusion, multiple imputations will be conducted, creating ten imputed data sets under the assumption of missingness at random. The result of the trial will be the pooled intervention effect and 95% CI of the analyses of the data sets after multiple imputations. The unadjusted, non-imputed analysis will also be made available.

**STATISTICAL PROCEDURES**

All analyses will be conducted by initially assigned study arm in an intention-to-treat analysis, and adjusted for randomization site. Thus, all randomized patients who will receive at least one dose of study treatment and who will have both a baseline and at least one post baseline measurement will be analyzed. The data will be expressed as mean ± SD. Statistical analyses will be conducted with SPSS version 19 (SPSS Institute, Chicago, Ill). Chi-square test will be done for categorical variables. T test will be done to assess the statistical significance of the continuous variables. Comparable nonparametric test (Mann-Whitney U test) will be substituted when tests for normality and equal variance failed. A value of P 0.05 will be used as a criterion for statistical significance. To test the effect of pycnogenol at defined time intervals, repeated measure test will be used

**MEASURES TO ADJUST FOR MULTIPLICITY, CONFOUNDERS, HETEROGENEITY, ETC.**

When the result of several outcomes is presented, Multiplicity may be a problem. We will consider adjustment with Bonferroni correction for conservative. For the secondary outcomes we have chosen to consider a *P* value below 0.01 as definitely statistical, whereas a *P* value between 0.01 and 0.05 will be considered indicative of statistical significance. *P* values above 0.05 will be considered as non-significant.

**QC PLANS**

The capsules will be given by the investigator to the patients by gavage, so fidelity to the intervention will be strong, however for more certainty, at the end of each day, the number of capsules remaining for each patient will be checked.

All staffs have the necessary skills at all stages of research, from conducting the study in ICU to conducting specialized tests of biomarkers and are approved by the University Scientific Committee.

**SAFETY OUTCOMES**

Adverse events are reported at each visit by nurse and physician. Concomitant medications Usage of medications during the study period will be recorded.

**PROGRAMMING PLANS**

Figure one will be a CONSORT flow chart. Figure two will be a bar chart with confidence intervals displaying the mean difference in inflammatory biomarkers in the two intervention and control groups. Figure three will be a bar chart with confidence intervals displaying the mean difference in oxidative stress biomarkers in the two intervention and control groups. Figure four will report outcome for mortality at 28 days

All tables will report variables according to randomization groups. Table one will report background variables. Table two will report changes in APACHE, SOFA and Nutric score. Table three will report changes in weight, body mass index and body fat percentage. Table four will report adverse events. Table six will report dietary intake. Table seven will report medications that used during intervention

**SENSITIVITY ANALYSES**

The study subjects with missing data on any of the variables will be excluded from the analysis. Analyses of these patients is seen as a sensitivity analysis to investigate whether conclusions are sensitive to assumptions regarding the pattern of missing data.
